# Discordant Expression of Circulating microRNA from Cellular and Extracellular Sources

**DOI:** 10.1371/journal.pone.0153691

**Published:** 2016-04-28

**Authors:** Ravi Shah, Kahraman Tanriverdi, Daniel Levy, Martin Larson, Mark Gerstein, Eric Mick, Joel Rozowsky, Robert Kitchen, Venkatesh Murthy, Ekaterina Mikalev, Jane E. Freedman

**Affiliations:** 1 Cardiovascular Institute, Beth Israel Deaconess Medical Center, Boston, MA, 02215, United States of America; 2 University of Massachusetts Medical School, Department of Medicine, Division of Cardiovascular Medicine, Worcester, MA, 01605, United States of America; 3 The Framingham Heart Study, Framingham, MA, United States of America and Population Sciences Branch, National Heart, Lung, and Blood Institute, National Institutes of Health, Bethesda, MD, United States of America; 4 Yale University Medical School, Computational Biology & Bioinformatics Program, New Haven, CT, 06520, United States of America; 5 University of Massachusetts Medical School, Department of Quantitative Health Sciences, Worcester, MA, 01605, United States of America; 6 Department of Cardiology, University of Michigan, Ann Arbor, Michigan, 1500 E. Medical Center Dr. SPC 5873, Ann Arbor, MI, 48109, United States of America; The University of Tokyo, JAPAN

## Abstract

MicroRNA (miRNA) expression has rapidly grown into one of the largest fields for disease characterization and development of clinical biomarkers. Consensus is lacking in regards to the optimal sample source or if different circulating sources are concordant. Here, using miRNA measurements from contemporaneously obtained whole blood- and plasma-derived RNA from 2391 individuals, we demonstrate that plasma and blood miRNA levels are divergent and may reflect different biological processes and disease associations.

## Introduction

MiRNAs are small RNAs that play an important role in the regulation of gene transcriptional networks. miRNAs are diverse in sequence and expression patterns and are evolutionarily conserved, suggesting they may participate in a wide range of genetic regulatory pathways[1] and pathophysiology[2, 3]. Given their role in direct gene regulation and accessibility in blood, intense interest has focused on the development and validation of miRNAs as diagnostic and prognostic biomarkers in human disease. Despite emerging data on disease-specific associations for various blood- and plasma-derived miRNAs, consensus is lacking as to whether a cellular, extracellular, or a mixed sample should be utilized to define disease mechanism and serve as biomarkers, and it remains unknown if expression from these distinct sources is concordant. Nevertheless, miRNA expression in translational human studies derived from whole blood (mostly cellular) or plasma (extracellular) is typically reported to be interchangeable. However, studies investigating more than one source of miRNA from a single donor are few[4–6] and direct comparisons in a large population with a large number of miRNAs are lacking[7].

To understand the comparability of cellular and extracellular sources of miRNA expression, we measured the levels of 159 microRNAs by high-throughput reverse transcription-quantitative polymerase chain reaction (RT-qPCR) isolated from both whole blood and plasma using samples obtained contemporaneously in 2,391 participants in the Framingham Heart Study (FHS). We hypothesized that expression of miRNAs in blood versus plasma would be discordant to support the notion that future studies in this field should be consistent in biofluids studied for translational biomarker development.

## Materials and Methods

### Study Population and Blood Collection

The Framingham Heart Study (FHS) Offspring Cohort is a community-based, prospective study of cardiovascular disease (CVD), with serial examinations every 4–8 years. Blood samples were collected during the 8^th^ Offspring Exam of the Framingham Heart Study (March 2005 –January 2008). Venipuncture was performed on study participants in a supine position, using standard venipuncture techniques. Blood was collected into blood collection tubes with a liquid buffered sodium citrate additive (0.105M). Blood collection tubes were centrifuged at 2,500g for 22 minutes at 4°C. Plasma was separated from the cells and frozen at -80°C within 90 minutes of draw. An aliquot of 170 μl of plasma samples was transferred to our laboratory in March 2014 and stored at -80°C. Plasma samples were centrifuged at 8,000g for 5 minutes immediately before RNA isolation.

Whole blood was collected in PAXgene (QIAGEN, Valencia, CA) tubes from each study participant after an overnight fast and stored at -80°C. The same laboratory, personnel and technical platform were used for measurement of plasma and whole blood miRNA. All participants gave written informed consent. The Boston University Medical Center Institutional Review Board approved FHS examination protocols and University of Massachusetts Medical School Review Board approved the study.

### miRNA Expression Profiling from Whole Blood

The high throughput Gene Expression and Biomarker Core Laboratory at the University of Massachusetts Medical School profiled 346 miRNAs isolated from whole blood in 2,445 FHS Offspring cohort participants using TaqMan miRNA assays. RNA isolation was performed by Asuragen, Inc (Austin, TX). Total RNA was isolated from the frozen PAXgene Blood RNA tubes. The initial miRNA list encompassed all TaqMan miRNA assays available at the start of the study. Isolated RNA samples were converted to complementary DNA using TaqMan miRNA Reverse Transcription Kit and MegaPlex Human RT Primer Pool Av2.1 and Pool Bv3.0. (Life Technologies, Foster City, CA, USA) in a 384-well Thermal Cycler. The complementary DNA samples were preamplified using TaqMan PreAmp Master Mix and PreAmp Primers, Human Pool A v2.1 and Pool B v3.0 (Life Technologies). Among 70 replicate samples, >95% of the data points had coefficients of variation <10% (mean ~4%). miRNA expression was quantified using quantification cycle (Cq), where higher Cq values reflect lower miRNA expression. We set miRNAs with Cq value ≤6 or ≥27 as missing for the subsequent whole blood miRNA analyses, as these values would indicate PCR results outside the linear range for the TaqMan chemistry used. By using Dynamic Arrays single copy can be detected in 26–27 cycles in TaqMan based qPCR analysis compare to 35–36 cycles in conventional plate-based qPCR analysis.

### miRNA Expression Profiling from Plasma

Of the 2,822 eligible subjects from FHS Offspring Exam 8, 59 (2%) subjects were excluded due to laboratory error (e.g., inaccurate volume of plasma pipetted, N = 31; poor protein precipitation performance, N = 23; or potential contamination, N = 5) resulting in 2,763 subjects as a final study cohort for plasma measurement. In total, expression of 331 human miRNAs was assayed from plasma. RNAs were reverse transcribed by using miScript II RT Kit (Cat. No: 218161, Qiagen, Fredrick, MD, USA). miScript Microfluidics PreAMP Kit (Cat. No: 331455, Qiagen, Fredrick, MD, USA) was used for preamplification reactions. Dynamic Array 96.96 GE (Fluidigm Corp., South San Francisco, CA) and Microfluidics qPCR Master Mix (Cat. No: 331431, Qiagen, Fredrick, MD, USA) with EvaGreen dye used for high-throughput qPCR reactions. miRNA assays were purchased from Qiagen in dried down format. PCR reactions were stopped at 23 cycles (Cq</ = 23). Similar to TaqMan chemistry, Dynamic Arrays used for high-throughput qPCR reactions provide single copy detection around 23 cycles of amplification in EvaGreen based qPCR analysis. Those plasma miRNAs with Cq value ≤6 or ≥23 cycles were considered outside the linear range for the Qiagen chemistry used, and were regarded as missing for the subsequent analyses.

### Statistical Methods

**Fig 1:**
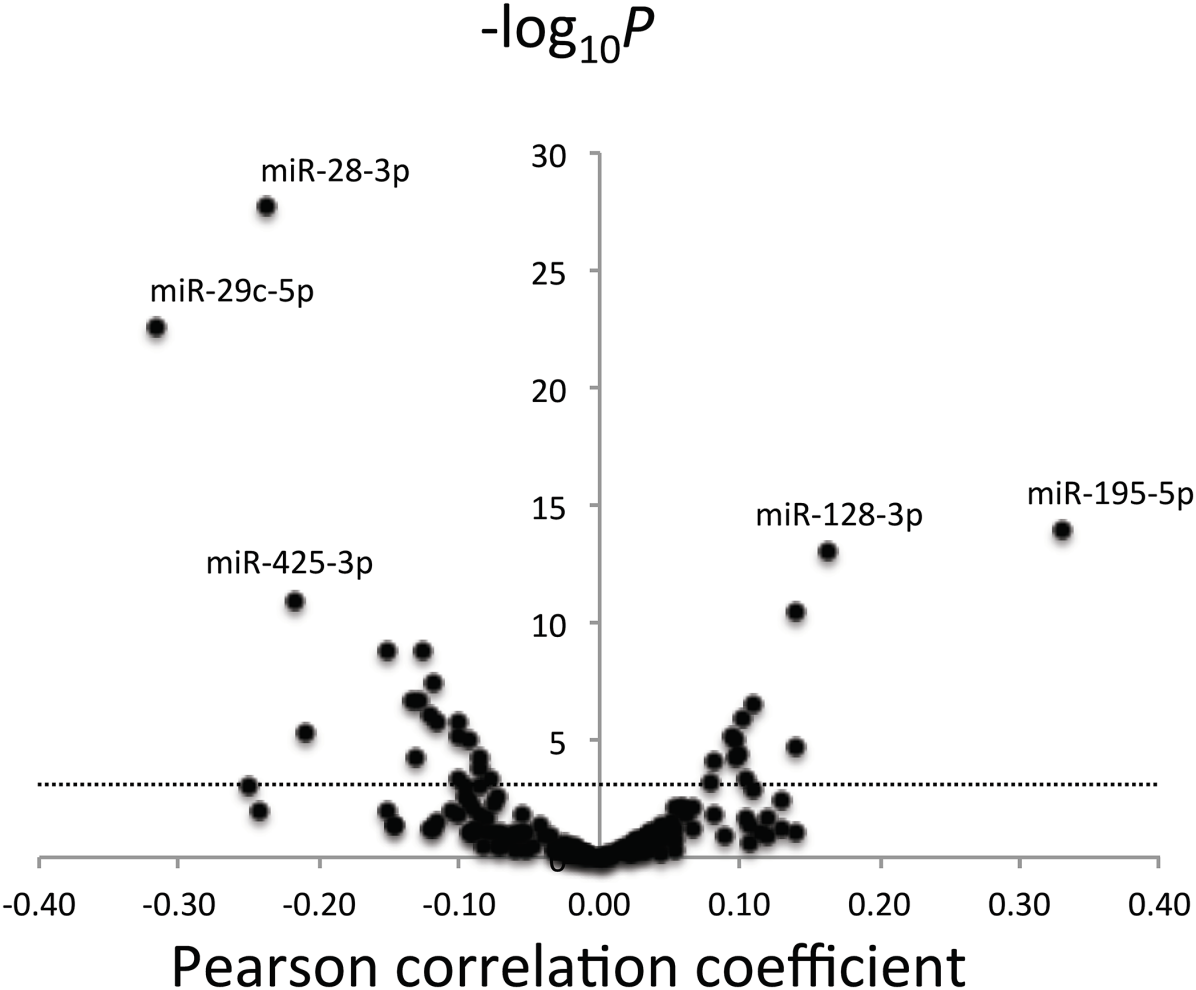
Volcano plot for Pearson correlations between whole blood and plasma miRNA PCR cycle values. Y-axes represent logarithm (base-10) of P value of each correlation coefficient. X-axes represent correlation coefficient. Dotted line is Bonferroni adjusted P value for 153 miRNA comparisons. Selected miRNAs are labeled on plots.

Only 153 miRNAs that were quantifiable in at least 100 FHS participants simultaneously in blood and plasma were included in this analysis. We performed both Spearman and Pearson bivariate correlation between whole blood- and plasma-derived miRNA expression (expressed as raw Cq), and constructed linear models for the association between blood and plasma miRNA expression (adjusted for age, sex, and several indices of RNA from the blood samples: RNA quality, A_260_/A_280_ ratio, and concentration). A Bonferroni-adjusted P value (for multiple comparisons) was used to assess significance. We constructed a volcano plot (with –log_10_(*p-value*) on the y-axis and magnitude of correlation on the x-axis) to display the distribution and significance of each bivariate association (Fig 1). SAS 9.3 (SAS Institute, Cary, NC) was used for all analyses.

## Results

Distribution of expression of all 153 miRNAs considered in this analysis in whole-blood and plasma is shown in S1 Table. The results of Spearman and Pearson correlation between miRNAs in whole blood- versus plasma are shown in Table 1 (for those miRNA correlations that were significant under a Bonferroni adjusted p-value 0.05/153≈0.0003 for Spearman, Pearson, or both) and Fig 1.

**Table 1:** Correlation coefficients (Pearson and Spearman) of whole blood vs. plasma miRNAs. *N* refers to the number of FHS participants on which each correlation is based. A P-value threshold (Bonferroni-adjusted) is specified in text.

miRNA	N	Spearman *ρ*	Spearman P	Pearson r	Pearson P
miR-28-3p	2099	-0.22	2.54x10^-24^	-0.24	1.99 x10^-28^
miR-29c-5p	937	-0.31	1.34 x10^-22^	-0.32	2.35 x10^-23^
miR-128	2058	0.20	2.45 x10^-20^	0.16	1.05 x10^-13^
miR-195-5p	517	0.33	1.18 x10^-14^	0.33	1.07 x10^-14^
miR-423-5p	1578	-0.18	1.80 x10^-13^	-0.16	8.74 x10^-11^
miR-30d-5p	1943	0.16	3.52 x10^-12^	0.13	5.25 x10^-9^
miR-19a-3p	2180	-0.14	8.71 x10^-11^	-0.12	3.85x10^-8^
miR-425-3p	960	-0.19	3.58 x10^-9^	-0.22	1.20 x10^-11^
miR-766-3p	1702	0.13	4.44 x10^-8^	0.10	5.45 x10^-5^
let-7a-5p	1581	-0.14	6.12 x10^-8^	-0.13	1.23 x10^-7^
miR-660-5p	936	-0.17	9.06 x10^-8^	-0.14	2.34 x10^-5^
miR-320b	1683	-0.13	1.51 x10^-7^	-0.12	1.33 x10^-6^
miR-103a-3p	1920	-0.12	1.54x10^-7^	-0.10	9.31 x10^-6^
miR-532-3p	1424	-0.13	3.28 x10^-7^	-0.12	2.24 x10^-6^
miR-151a-5p	2092	0.11	6.16x10^-7^	0.10	9.46 x10^-6^
miR-494	907	0.16	1.82 x10^-6^	0.14	2.19 x10^-5^
miR-148b-3p	1607	-0.11	4.48x10^-6^	-0.12	1.02x10^-6^
miR-29c-3p	2217	0.10	4.51x10^-6^	0.10	1.35x10^-6^
miR-320a	2228	-0.09	2.06x10^-5^	-0.10	6.84x10^-6^
miR-652-3p	1709	0.10	4.37x10^-5^	0.09	0.0001
miR-1260a	2190	-0.08	0.0001	-0.10	1.90x10^-6^
miR-27a-3p	2221	-0.08	0.0002	-0.08	6.21x10^-5^
miR-324-3p	1121	0.17	1.22x10^-8^	0.10	0.0005
miR-532-5p	1187	-0.14	1.06x10^-6^	-0.10	0.0005
miR-199a-3p	1463	-0.11	3.33x10^-5^	-0.09	0.001
miR-27b-3p	1965	-0.09	0.0001	-0.08	0.0005
miR-15b-5p	1809	-0.07	0.002	-0.09	0.0002
miR-574-3p	1931	0.07	0.003	0.08	0.0002
miR-625-3p	468	-0.13	0.006	-0.24	1.09x10^-7^
miR-374b-5p	1213	-0.08	0.006	-0.11	0.0001

Given the general concordance between Pearson and Spearman correlations, we constructed linear models for the association between whole blood and plasma miRNA expression (in raw C_q_ level adjusted for age, sex, and several indices of RNA from the whole blood samples: RNA quality, A_260_/A_280_ ratio, and concentration) for those miRNAs in Table 1. These results are shown in Table 2 (with threshold P-value based on 30 comparisons: 0.05/30≈0.0017).

**Table 2:** Estimated regression coefficients from linear models of whole blood (independent) vs. plasma miRNAs (dependent variable in regression). Each model was adjusted for age, sex, and RNA characteristics as specified in text. A P-value threshold (Bonferroni-adjusted) is specified in text.

miRNA	Estimated regression coefficient (adjusted)	P-value
let-7a-5p	-0.055	2.46x10^-8^
miR-103a-3p	-0.051	1.85 x10^-6^
miR-1260a	-0.070	1.84 x10^-7^
miR-128-3p	0.129	3.33 x10^-13^
miR-148b-3p	-0.079	6.80 x10^-9^
miR-151a-5p*	0.050	0.004
miR-15b-5p	-0.049	8.49x10^-6^
miR-195-5p	0.319	3.74x10^-13^
miR-199a-3p	-0.045	0.0009
miR-19a-3p	-0.065	1.26x10^-10^
miR-27a-3p	-0.086	2.87x10^-7^
miR-27b-3p	-0.059	4.50x10^-5^
miR-28-3p	-0.173	1.07x10^-29^
miR-29c-3p	0.025	0.002
miR-29c-5p	-0.183	6.24x10^-16^
miR-30d-5p	0.076	4.48x10^-7^
miR-320a	-0.066	8.65x10^-5^
miR-320b	-0.143	0.0004
miR-324-3p*	0.086	0.004
miR-374b-5p	-0.043	5.98x10^-5^
miR-423-5p	-0.093	7.18x10^-11^
miR-425-3p	-0.072	0.0004
miR-494-3p	0.114	0.0002
miR-532-3p	-0.102	3.93x10^-6^
miR-532-5p	-0.081	0.0004
miR-574-3p	0.031	0.005
miR-625-3p	-0.111	4.16x10^-5^
miR-652-3p	0.126	1.23x10^-5^
miR-660-5p	-0.064	0.0005
miR-766-3p	0.087	7.40x10^-6^

Despite several miRNAs reaching statistical significance, the directionality of correlation between plasma and whole blood miRNAs was not consistent.

## Discussion

These findings are relevant for design of experiments and clinical studies involving miRNAs as clinical biomarkers in human disease[8–10]. Both plasma and whole blood contain circulating RNAs[11, 12], and circulating transcripts from cellular sources (e.g., platelets) are associated with cardiovascular disease and its risk factors[13, 14]. In addition, cellular-derived particles (e.g., from platelets[15]) have been shown to transfer genetic information, regulate gene expression at a distance, and are actively being investigated as disease therapeutics[15]. The emerging role of plasma- and cellular-derived RNAs as disease reporters and therapeutics makes standardization of the source of blood for miRNA assay critical.

While technical comparisons have been reported[5], whether different circulating sources (e.g., plasma vs. whole blood) are comparable in the same individual is unclear, and as such, miRNA results for both whole blood and plasma are routinely reported[7]. Despite long-term stability of expression in both compartments[16], whole blood has a variety of cellular subtypes that harbor RNA (e.g., platelet, erythrocyte, and inflammatory white blood cells) and high levels of whole blood-based cellular miRNAs may mask disease-specific expression[7] that may not be the case with plasma-derived RNAs. On the other hand, for miRNAs with local intracellular function in a given disease plasma expression may not be as informative.

Although there have been statements about differences and need for comparisons[7], direct data are lacking. A limited comparison of bovine blood and plasma by RNA sequencing suggests similarities in expression [17], leading to an assumption that they are comparable, possibly due to release of protein-bound small RNAs or exosomes. However, in a recent analysis from our group of 13 patients with cardiovascular disease, miRNA profiles were distinct between whole blood and plasma[18]. Similar results were found in 9 women followed during a menstrual cycle with variability noted between plasma and whole blood miRNA expression[19]. Our study provides the first, large-scale demonstration that whole blood and plasma miRNA expression is distinct, and calls for the standardization of measurement sources for clinical and translational research involving extracellular RNA.

The results of our study should be viewed in the context of its design. A major strength of our study is its use of a large, community-based cohort with standard techniques (e.g., RT-qPCR) to assay expression of a wide array of miRNAs from concomitantly obtained blood samples. One important limitation is the use of different RNA isolation methods and PCR reagents. While different RNA isolation methods and PCR reagents (e.g., TaqMan versus Qiagen) could potentially lead to differences in yield (even based on miRNA species), we attempted to account for characteristics of whole blood-derived RNA quality in regression models and had a high sample size relative to existing studies of miRNAs in blood and plasma. Nevertheless, direct comparisons across RNA isolation kits, PCR methodology, and biofluids are a subject of ongoing investigation in multiple groups and will further inform heterogeneity on the basis of these variables. miRNA profiling was performed from plasma or whole blood samples and as such, does not provide information about compartments for RNA, including exosomes, protein- or lipid-bound RNA, or free circulating fraction. While RNA sequencing methods would be more sensitive across a wider array of miRNAs and for other small and long non-coding RNAs, RT-qPCR is a well-validated method with facile clinical translation for the most widely studied extracellular RNAs to date (miRNAs). Finally, though hemolysis may affect miRNA abundance in plasma, we observed that differences in Cq value between miR-23a (in variant with hemolysis) and miR-451 (a red blood cell marker) was not elevated (>7) in the majority of samples (99%), suggesting an absence of hemolysis contributing to results.

In summary, we compared expression of miRNAs measured by high-throughput RT-qPCR isolated from both whole blood and plasma using contemporaneously samples in 2,391 individuals in the community. There was only modest association between cellular and extracellular miRNA expression in this large study sample. These results demonstrate for the first time in a large human cohort that miRNA expression from cellular versus acellular sources may be divergent, and point to care in designing clinical studies to validate known and novel extracellular RNA markers in human disease. miRNA expression from different human sources (e.g., whole blood, plasma) should not be used interchangeably as biomarkers of disease in translational investigation.

## Supporting Information

S1 Table Summary characteristics of 153 miRNAs assayed in whole blood and plasma in FHS participants. The numbers of subjects in this table (N) correspond to the number of subjects with measured values for each miRNA in blood or plasma (but do not indicate the number of subjects in common).
(DOCX)
