# Supplementary material for: Discordant Expression of Circulating microRNA from Cellular and Extracellular Sources
Source: PLoS One. 2016 Apr 28;11(4):e0153691. doi: 10.1371/journal.pone.0153691 (PMC4849639; doi:10.1371/journal.pone.0153691)
Supplement: S1 Table — The numbers of subjects in this table (N) correspond to the number of subjects with measured values for each miRNA in blood or plasma (but do not indicate the number of subjects in common). (DOCX) [file pone.0153691.s001.docx]

| **miRNA** | Whole Blood | | | | Plasma | | | |
| --- | --- | --- | --- | --- | --- | --- | --- | --- |
|  | **N** | **Median** | **25%tile** | **75%tile** | **N** | **Median** | **25%tile** | **75%tile** |
| **let-7a-5p** | 1889 | 19.7 | 17.4 | 22.3 | 1993 | 19.4 | 18.6 | 20.3 |
| **let-7b-5p** | 2036 | 15.9 | 14.7 | 17.3 | 1980 | 19.5 | 18.6 | 20.4 |
| **let-7c** | 2036 | 17.8 | 15.9 | 20.0 | 1727 | 20.2 | 19.5 | 20.9 |
| **let-7d-3p** | 915 | 22.4 | 20.4 | 24.3 | 1838 | 19.9 | 19.2 | 20.6 |
| **let-7d-5p** | 420 | 15.2 | 14.2 | 16.2 | 1936 | 19.6 | 18.7 | 20.4 |
| **let-7e-5p** | 2285 | 15.6 | 14.1 | 17.3 | 1128 | 19.1 | 18.1 | 20.1 |
| **let-7g-5p** | 2358 | 14.6 | 13.5 | 16.1 | 1907 | 19.5 | 18.6 | 20.4 |
| **miR-100-5p** | 1953 | 17.5 | 15.3 | 22.1 | 1153 | 18.9 | 18.2 | 20.0 |
| **miR-103a-3p** | 2360 | 15.0 | 13.8 | 16.4 | 1948 | 19.6 | 18.8 | 20.5 |
| **miR-106b-3p** | 2317 | 13.4 | 12.4 | 14.6 | 1646 | 18.2 | 16.6 | 19.1 |
| **miR-106b-5p** | 1686 | 8.1 | 6.9 | 10.4 | 2259 | 17.7 | 16.9 | 18.5 |
| **miR-10a-5p** | 688 | 23.3 | 21.6 | 25.3 | 706 | 20.9 | 20.4 | 21.5 |
| **miR-1180** | 2359 | 16.3 | 15.6 | 17.2 | 1036 | 19.7 | 19.1 | 20.3 |
| **miR-125a-5p** | 2350 | 15.0 | 13.8 | 16.4 | 2033 | 19.4 | 18.6 | 20.2 |
| **miR-125b-5p** | 2376 | 12.6 | 10.8 | 14.1 | 2017 | 19.8 | 19.0 | 20.5 |
| **miR-126-3p** | 2216 | 9.3 | 8.0 | 10.8 | 2311 | 16.1 | 15.3 | 16.7 |
| **miR-126-5p** | 1878 | 22.0 | 19.7 | 24.2 | 2335 | 16.7 | 16.0 | 17.4 |
| **miR-1260a** | 2356 | 17.5 | 16.3 | 18.8 | 2224 | 18.3 | 17.6 | 19.3 |
| **miR-128** | 2351 | 16.1 | 15.3 | 17.1 | 2091 | 19.2 | 18.2 | 20.1 |
| **miR-130a-3p** | 2125 | 13.2 | 11.9 | 15.7 | 2055 | 19.5 | 18.7 | 20.3 |
| **miR-130b-3p** | 2282 | 16.8 | 15.5 | 19.3 | 973 | 18.9 | 18.2 | 19.8 |
| **miR-130b-5p** | 2304 | 19.8 | 18.9 | 20.7 | 441 | 20.9 | 20.2 | 21.4 |
| **miR-132-3p** | 2324 | 14.8 | 13.7 | 16.3 | 722 | 21.0 | 20.4 | 21.6 |
| **miR-133a** | 2319 | 15.9 | 14.1 | 17.7 | 499 | 20.9 | 20.3 | 21.5 |
| **miR-139-5p** | 2363 | 10.9 | 9.8 | 12.4 | 1556 | 20.1 | 19.4 | 20.8 |
| **miR-140-3p** | 2341 | 11.3 | 10.3 | 12.8 | 2164 | 19.2 | 18.5 | 20.0 |
| **miR-142-3p** | 2346 | 14.0 | 12.5 | 15.8 | 879 | 19.7 | 18.9 | 20.5 |
| **miR-142-5p** | 1878 | 22.8 | 21.2 | 24.5 | 2173 | 18.7 | 17.8 | 19.6 |
| **miR-144-5p** | 2287 | 15.9 | 13.8 | 18.3 | 798 | 20.9 | 20.4 | 21.5 |
| **miR-145-5p** | 2355 | 11.6 | 10.1 | 13.9 | 2146 | 19.5 | 18.7 | 20.2 |
| **miR-146a-5p** | 2227 | 11.9 | 10.4 | 14.2 | 2264 | 18.4 | 17.7 | 19.2 |
| **miR-146b-5p** | 2329 | 12.7 | 11.6 | 14.1 | 1313 | 20.3 | 19.4 | 21.0 |
| **miR-148a-3p** | 1445 | 21.2 | 19.4 | 23.7 | 2215 | 18.2 | 17.3 | 19.3 |
| **miR-148b-3p** | 1744 | 21.7 | 20.0 | 23.9 | 2208 | 18.5 | 17.6 | 19.5 |
| **miR-151a-3p** | 2351 | 12.8 | 11.7 | 14.0 | 1001 | 18.8 | 18.0 | 19.7 |
| **miR-151a-5p** | 2349 | 12.9 | 12.0 | 14.0 | 2129 | 19.4 | 18.6 | 20.2 |
| **miR-152** | 2343 | 16.6 | 15.3 | 18.2 | 768 | 20.5 | 19.9 | 21.2 |
| **miR-155-5p** | 2105 | 18.7 | 17.1 | 20.9 | 431 | 21.2 | 20.6 | 21.6 |
| **miR-15b-3p** | 2354 | 11.2 | 10.2 | 12.5 | 781 | 20.6 | 20.0 | 21.2 |
| **miR-15b-5p** | 1901 | 8.0 | 7.2 | 9.8 | 2291 | 16.9 | 16.2 | 17.6 |
| **miR-16-5p** | 2067 | 9.0 | 7.6 | 11.4 | 2351 | 13.5 | 12.7 | 14.2 |
| **miR-17-5p** | 2316 | 9.8 | 8.5 | 11.4 | 2260 | 17.8 | 17.0 | 18.6 |
| **miR-181a-2-3p** | 2322 | 15.2 | 14.3 | 16.3 | 893 | 20.0 | 19.0 | 20.8 |
| **miR-181a-5p** | 2194 | 16.4 | 15.3 | 17.8 | 686 | 20.9 | 20.4 | 21.5 |
| **miR-182-5p** | 1910 | 21.9 | 19.4 | 24.2 | 266 | 21.2 | 20.7 | 21.6 |
| **miR-185-5p** | 2335 | 9.8 | 8.5 | 11.4 | 2122 | 19.1 | 18.2 | 19.9 |
| **miR-192-5p** | 2317 | 10.2 | 8.5 | 12.0 | 1762 | 20.0 | 19.2 | 20.7 |
| **miR-193a-5p** | 2355 | 16.7 | 15.9 | 17.8 | 603 | 19.6 | 17.1 | 21.1 |
| **miR-193b-3p** | 2337 | 18.5 | 16.9 | 20.6 | 631 | 20.0 | 18.5 | 21.1 |
| **miR-194-5p** | 419 | 10.9 | 10.0 | 11.8 | 2094 | 18.2 | 17.2 | 19.7 |
| **miR-195-5p** | 539 | 22.4 | 20.3 | 24.8 | 2238 | 17.2 | 13.5 | 19.4 |
| **miR-197-3p** | 2359 | 11.8 | 11.1 | 12.9 | 2069 | 19.9 | 19.3 | 20.6 |
| **miR-199a-3p** | 1582 | 23.6 | 21.7 | 25.0 | 2221 | 18.6 | 17.8 | 19.5 |
| **miR-199a-5p** | 1181 | 24.0 | 22.2 | 25.5 | 831 | 19.6 | 18.8 | 20.5 |
| **miR-19a-3p** | 2286 | 17.4 | 15.9 | 19.7 | 2283 | 15.8 | 14.9 | 16.5 |
| **miR-19b-3p** | 639 | 7.1 | 6.5 | 8.1 | 2301 | 15.0 | 14.3 | 15.8 |
| **miR-200c-3p** | 1804 | 22.9 | 21.4 | 24.5 | 348 | 21.0 | 20.5 | 21.5 |
| **miR-204-5p** | 528 | 24.8 | 23.1 | 25.9 | 1456 | 8.7 | 7.7 | 9.4 |
| **miR-206** | 2342 | 13.0 | 11.8 | 14.1 | 339 | 21.3 | 20.6 | 21.7 |
| **miR-20a-5p** | 1992 | 8.4 | 7.2 | 9.9 | 2281 | 16.8 | 16.1 | 17.6 |
| **miR-20b-5p** | 2356 | 12.7 | 11.6 | 14.1 | 1033 | 16.8 | 16.0 | 17.5 |
| **miR-212-3p** | 2025 | 21.4 | 19.8 | 23.5 | 960 | 20.1 | 19.4 | 20.9 |
| **miR-22-3p** | 2289 | 16.3 | 15.0 | 18.1 | 2277 | 16.4 | 15.7 | 17.3 |
| **miR-22-5p** | 2306 | 17.2 | 16.4 | 18.2 | 873 | 20.6 | 19.9 | 21.1 |
| **miR-221-3p** | 2306 | 14.2 | 13.2 | 15.5 | 2297 | 17.7 | 17.0 | 18.5 |
| **miR-222-3p** | 2332 | 12.5 | 10.8 | 14.8 | 2192 | 19.2 | 18.4 | 19.9 |
| **miR-223-5p** | 2171 | 20.5 | 19.4 | 21.8 | 636 | 21.0 | 20.4 | 21.5 |
| **miR-224-5p** | 1330 | 23.7 | 21.9 | 25.2 | 646 | 20.9 | 20.3 | 21.4 |
| **miR-24-2-5p** | 2061 | 20.7 | 19.3 | 22.3 | 249 | 21.2 | 20.6 | 21.7 |
| **miR-24-3p** | 2017 | 7.3 | 6.7 | 8.5 | 2292 | 16.5 | 15.8 | 17.2 |
| **miR-25-3p** | 558 | 7.2 | 6.7 | 7.9 | 2304 | 16.9 | 16.1 | 17.8 |
| **miR-26a-5p** | 2320 | 10.3 | 9.2 | 11.7 | 2299 | 17.3 | 16.5 | 18.1 |
| **miR-26b-5p** | 2305 | 16.3 | 14.5 | 18.6 | 2296 | 17.4 | 16.5 | 18.2 |
| **miR-27a-3p** | 2355 | 15.9 | 14.7 | 17.5 | 2257 | 17.2 | 16.3 | 18.6 |
| **miR-27b-3p** | 2337 | 18.2 | 17.1 | 19.4 | 2002 | 19.1 | 18.2 | 20.1 |
| **miR-28-3p** | 2353 | 16.2 | 15.0 | 18.1 | 2134 | 17.4 | 16.1 | 18.6 |
| **miR-28-5p** | 2355 | 11.5 | 10.4 | 13.8 | 1768 | 19.6 | 18.7 | 20.6 |
| **miR-296-5p** | 2326 | 12.4 | 11.3 | 13.8 | 593 | 21.1 | 20.5 | 21.6 |
| **miR-29a-3p** | 2277 | 18.4 | 16.6 | 20.4 | 2277 | 16.8 | 16.1 | 17.6 |
| **miR-29c-3p** | 2334 | 14.2 | 11.9 | 17.0 | 2274 | 16.8 | 16.1 | 17.6 |
| **miR-29c-5p** | 2298 | 20.6 | 19.4 | 21.9 | 983 | 20.3 | 19.1 | 21.1 |
| **miR-301a-3p** | 1909 | 22.5 | 20.6 | 24.2 | 761 | 20.8 | 20.2 | 21.4 |
| **miR-30a-3p** | 880 | 23.4 | 21.6 | 24.9 | 1326 | 17.6 | 16.7 | 19.1 |
| **miR-30a-5p** | 625 | 7.7 | 6.8 | 8.9 | 2313 | 16.9 | 16.2 | 17.5 |
| **miR-30b-5p** | 562 | 6.9 | 6.4 | 7.5 | 2018 | 19.7 | 18.9 | 20.4 |
| **miR-30d-5p** | 2028 | 7.8 | 6.9 | 9.5 | 2294 | 17.2 | 16.4 | 18.0 |
| **miR-30e-3p** | 2346 | 11.8 | 11.0 | 12.9 | 187 | 20.9 | 20.2 | 21.5 |
| **miR-320a** | 2333 | 8.8 | 8.0 | 9.8 | 2285 | 18.5 | 17.8 | 19.3 |
| **miR-320b** | 2369 | 8.7 | 8.1 | 9.8 | 1701 | 16.4 | 14.3 | 19.9 |
| **miR-323a-3p** | 775 | 24.9 | 23.8 | 25.8 | 742 | 20.9 | 20.3 | 21.5 |
| **miR-324-3p** | 2342 | 10.0 | 9.1 | 11.2 | 1149 | 17.8 | 17.3 | 18.8 |
| **miR-324-5p** | 2323 | 12.6 | 11.5 | 14.1 | 1315 | 20.3 | 19.6 | 20.8 |
| **miR-329** | 1555 | 21.9 | 20.5 | 23.5 | 1112 | 20.6 | 19.9 | 21.2 |
| **miR-330-3p** | 1828 | 17.6 | 16.6 | 19.1 | 153 | 21.2 | 20.7 | 21.7 |
| **miR-331-3p** | 1032 | 7.0 | 6.5 | 7.8 | 883 | 20.9 | 20.3 | 21.5 |
| **miR-335-5p** | 1476 | 21.1 | 19.5 | 23.6 | 1181 | 20.8 | 20.3 | 21.4 |
| **miR-339-3p** | 2350 | 17.1 | 15.9 | 18.5 | 130 | 21.0 | 20.5 | 21.6 |
| **miR-339-5p** | 2340 | 10.7 | 9.5 | 12.0 | 963 | 20.0 | 19.4 | 20.6 |
| **miR-340-5p** | 725 | 24.3 | 22.8 | 25.6 | 1278 | 20.5 | 19.8 | 21.1 |
| **miR-342-3p** | 2318 | 8.8 | 7.8 | 10.0 | 2320 | 17.8 | 17.1 | 18.4 |
| **miR-342-5p** | 574 | 24.2 | 22.6 | 25.5 | 374 | 21.0 | 20.4 | 21.5 |
| **miR-345-5p** | 2354 | 16.5 | 15.4 | 17.9 | 499 | 21.0 | 20.3 | 21.5 |
| **miR-361-5p** | 1961 | 22.0 | 20.4 | 23.6 | 745 | 20.8 | 20.1 | 21.3 |
| **miR-363-3p** | 2323 | 12.3 | 11.1 | 14.1 | 1764 | 19.4 | 18.7 | 20.4 |
| **miR-365a-3p** | 1555 | 21.9 | 20.6 | 23.9 | 1263 | 20.5 | 19.8 | 21.1 |
| **miR-374a-5p** | 2327 | 16.3 | 14.7 | 18.3 | 744 | 20.1 | 19.3 | 20.8 |
| **miR-374b-5p** | 2331 | 17.6 | 16.1 | 19.6 | 1251 | 20.5 | 19.8 | 21.1 |
| **miR-375** | 2268 | 20.8 | 19.8 | 22.0 | 815 | 20.4 | 19.8 | 21.1 |
| **miR-376a-3p** | 2114 | 17.3 | 15.3 | 19.3 | 547 | 21.0 | 20.4 | 21.6 |
| **miR-376c** | 846 | 24.4 | 22.7 | 25.6 | 1205 | 20.5 | 19.8 | 21.2 |
| **miR-378a-3p** | 323 | 19.4 | 18.0 | 21.4 | 671 | 20.5 | 19.9 | 21.1 |
| **miR-378a-5p** | 2363 | 17.6 | 16.8 | 18.6 | 444 | 20.9 | 20.4 | 21.5 |
| **miR-409-3p** | 2357 | 10.5 | 9.1 | 11.9 | 420 | 20.6 | 19.9 | 21.4 |
| **miR-423-5p** | 1783 | 14.8 | 13.3 | 16.0 | 2120 | 19.3 | 18.5 | 20.2 |
| **miR-424-3p** | 2286 | 19.0 | 18.3 | 19.9 | 111 | 21.3 | 20.2 | 21.6 |
| **miR-425-3p** | 2301 | 20.4 | 18.4 | 22.2 | 1003 | 19.8 | 18.8 | 20.4 |
| **miR-432-5p** | 823 | 24.1 | 22.1 | 25.6 | 1292 | 20.3 | 19.5 | 21.0 |
| **miR-433** | 1096 | 24.7 | 23.5 | 25.7 | 1541 | 20.5 | 19.9 | 21.1 |
| **miR-451a** | 456 | 7.0 | 6.5 | 7.9 | 2304 | 11.8 | 10.8 | 13.0 |
| **miR-483-3p** | 784 | 23.8 | 22.3 | 25.2 | 691 | 20.9 | 20.4 | 21.5 |
| **miR-483-5p** | 841 | 16.4 | 12.2 | 21.8 | 880 | 20.0 | 18.6 | 20.9 |
| **miR-484** | 169 | 6.4 | 6.2 | 6.9 | 2280 | 17.9 | 17.1 | 18.6 |
| **miR-486-3p** | 1293 | 21.1 | 19.6 | 23.0 | 699 | 20.8 | 20.2 | 21.4 |
| **miR-487b** | 493 | 24.7 | 23.5 | 25.7 | 529 | 20.8 | 20.1 | 21.5 |
| **miR-494** | 1176 | 23.2 | 21.4 | 25.0 | 1632 | 16.3 | 15.3 | 18.5 |
| **miR-500a-3p** | 2039 | 23.0 | 22.1 | 24.1 | 315 | 21.2 | 20.5 | 21.6 |
| **miR-505-3p** | 1872 | 22.8 | 21.4 | 24.1 | 688 | 20.8 | 20.0 | 21.3 |
| **miR-532-3p** | 2103 | 8.5 | 7.5 | 9.6 | 1668 | 19.6 | 18.5 | 20.8 |
| **miR-532-5p** | 2210 | 14.2 | 13.0 | 15.7 | 1308 | 19.9 | 18.2 | 20.9 |
| **miR-542-3p** | 318 | 24.2 | 23.0 | 25.5 | 983 | 17.9 | 17.3 | 18.7 |
| **miR-543** | 2086 | 22.3 | 20.8 | 24.0 | 311 | 20.8 | 20.0 | 21.4 |
| **miR-574-3p** | 2329 | 9.9 | 8.9 | 11.4 | 1983 | 19.7 | 18.7 | 20.5 |
| **miR-589-5p** | 1641 | 24.0 | 22.8 | 25.3 | 730 | 20.6 | 20.0 | 21.1 |
| **miR-590-5p** | 1966 | 17.6 | 14.8 | 20.7 | 600 | 20.5 | 19.8 | 21.1 |
| **miR-598** | 2114 | 22.8 | 21.4 | 24.3 | 139 | 21.2 | 20.5 | 21.6 |
| **miR-616-5p** | 2259 | 20.3 | 19.3 | 21.7 | 1135 | 17.4 | 16.7 | 18.2 |
| **miR-625-3p** | 2317 | 15.1 | 13.6 | 16.8 | 487 | 20.8 | 20.1 | 21.5 |
| **miR-628-3p** | 2330 | 20.1 | 19.2 | 21.2 | 1032 | 18.6 | 17.9 | 19.3 |
| **miR-642a-5p** | 2344 | 13.3 | 12.4 | 14.6 | 912 | 19.8 | 18.9 | 20.5 |
| **miR-652-3p** | 2273 | 10.4 | 9.5 | 11.8 | 1801 | 19.6 | 16.9 | 20.5 |
| **miR-654-5p** | 1850 | 20.9 | 19.6 | 22.8 | 179 | 21.2 | 20.6 | 21.7 |
| **miR-660-5p** | 2354 | 15.7 | 14.4 | 17.1 | 960 | 20.2 | 19.4 | 20.9 |
| **miR-7-1-3p** | 2308 | 13.3 | 11.3 | 15.8 | 188 | 21.0 | 19.8 | 21.6 |
| **miR-7-5p** | 2273 | 19.0 | 17.7 | 20.5 | 180 | 21.0 | 20.4 | 21.6 |
| **miR-744-5p** | 2296 | 11.8 | 10.9 | 13.0 | 1607 | 12.5 | 11.5 | 15.5 |
| **miR-766-3p** | 2361 | 8.9 | 8.2 | 9.9 | 1726 | 19.5 | 18.9 | 20.4 |
| **miR-769-5p** | 2319 | 18.7 | 17.7 | 19.9 | 1035 | 20.7 | 20.1 | 21.3 |
| **miR-885-5p** | 2244 | 19.7 | 18.4 | 21.6 | 1632 | 19.9 | 18.8 | 20.7 |
| **miR-9-3p** | 1777 | 23.0 | 21.7 | 24.5 | 243 | 21.2 | 20.7 | 21.6 |
| **miR-93-3p** | 2355 | 9.1 | 8.2 | 10.1 | 362 | 21.2 | 20.6 | 21.7 |
| **miR-93-5p** | 2329 | 10.0 | 8.9 | 11.2 | 2263 | 17.9 | 17.1 | 18.6 |
| **miR-941** | 1189 | 24.4 | 23.0 | 25.6 | 1171 | 18.7 | 18.1 | 19.6 |
| **miR-99b-5p** | 2369 | 11.9 | 10.8 | 13.5 | 1901 | 19.9 | 19.0 | 20.7 |
